# Supplementary material for: Quantitative Mass Spectrometry Characterizes Client Spectra of Components for Targeting of Membrane Proteins to and Their Insertion into the Membrane of the Human ER
Source: Int J Mol Sci. 2023 Sep 15;24(18):14166. doi: 10.3390/ijms241814166 (PMC10532041; doi:10.3390/ijms241814166)
Supplement: Supplementary file 1 [file ijms-24-14166-s001.zip › ijms-2576814-supplementary.pdf]

Table S1

MS run / Sample number	Project title / Sample description	Dataset identifier	Reference
**Orbi1763**	**Clients of human TRAP complex**	**PXD008178**	
Sample 1-3	scr control siRNA		Nguyen et al., 2018
Sample 4-6	SEC61A1 siRNA #4		Nguyen et al., 2018
Sample 7-9	SEC61A1 siRNA #5		Nguyen et al., 2018
**Orbi1931**	**Clients of human TRAP complex**	**PXD008178**	
Sample 1-3	scr control siRNA		
Sample 4-6	SEC63 siRNA #5		Schorr et al., 2019
Sample 7-9	SEC63 siRNA #7		Schorr et al., 2019
Sample 10-12	TRAPB siRNA #2		Nguyen et al., 2018
Sample 13-15	TRAPB siRNA #3		Nguyen et al., 2018
Sample 16-18	scr control siRNA		
Sample 19-21	SEC62 siRNA #2		Schorr et al., 2019
Sample 22-24	SEC62 siRNA UTR		Schorr et al., 2019
Sample 25-27	TRAM siRNA #2		Klein et al., 2020
Sample 28-30	TRAM siRNA #6		Klein et al., 2020
**Orbi1986**	**Clients of human TRAP complex**	**PXD008178**	
Sample 1-3	scr control siRNA		
Sample 4-6	SRA siRNA #3		Tirincsi et al., 2022
Sample 7-9	SRA siRNA #6		Tirincsi et al., 2022
Sample 10-12	TRAP siRNA #2		Nguyen et al., 2018
Sample 13-15	TRAP siRNA #3		Nguyen et al., 2018
**Orbi2048**	**Targeting of precursor polypeptides to the human ER**	**PXD008178**	
Sample 1-3	scr control siRNA		
Sample 4-6	BIP siRNA CA		Schorr et al., 2019
Sample 7-9	BIP siRNA UTR#1		Schorr et al., 2019
Sample 10-12	SEC61A1 siRNA #4		Nguyen et al., 2018
Sample 13-15	SEC61A1 siRNA #5		Nguyen et al., 2018
Sample 16-18	scr control siRNA		
Sample 19-21	WRB siRNA #3		Tirincsi et al., 2022
Sample 22-24	WRB siRNA #4		Tirincsi et al., 2022
Sample 25-27	WRB siRNA #4 + SND2 siRNA #2 = WS		unpublished
Sample 28-30	WRB siRNA #4 + SND2 siRNA #3 = WS		unpublished
Sample 31	control fibroblasts #1		Nguyen et al., 2018
Sample 32	control fibroblasts #2		Nguyen et al., 2018
Sample 33	CDG patient fibroblasts DSSR3		Nguyen et al., 2018
Sample 34	CDG patient fibroblasts DSSR4 #1		Nguyen et al., 2018
Sample 35	CDG patient fibroblasts DSSR4 #2		Nguyen et al., 2018
**Orbi2085**	**Clients of human TRAP complex**	**PXD008178**	
Sample 1-3	scr control siRNA		
Sample 4-6	TRAP siRNA #2		Nguyen et al., 2018
Sample 7-9	TRAP siRNA #3		Nguyen et al., 2018
Sample 10-12	TRAM siRNA #2		Klein et al., 2020
Sample 13-15	TRAM siRNA #6		Klein et al., 2020
**Orbi2155**	**Clients of human Sec62/Sec63 complex**	**PXD012078**	
Sample 1-3	scr control siRNA		
Sample 4-6	SEC62 siRNA #2		Schorr et al., 2019
Sample 7-9	SEC62 UTR siRNA		Schorr et al., 2019
Sample 10-12	SRA siRNA #3		Tirincsi et al., 2022
Sample 13-15	SRA siRNA #6		Tirincsi et al., 2022

MS run	Project title	Dataset identifier	Reference
Sample number	Sample description		
**Orbi2288**	**mRNA targeting to the human ER**	**PXD011989**	
Sample 1-3	scr control siRNA		Bhadra et al., 2021
Sample 4-6	P180 siRNA #1		Bhadra et al., 2021
Sample 7-9	P180 siRNA #2		Bhadra et al., 2021
**Orbi2314**	**mRNA targeting to the human ER**	**PXD011990**	Bhadra et al., 2021
Sample 1-3	scr control siRNA		
Sample 4-6	KTN1 siRNA #3		Bhadra et al., 2021
Sample 7-9	KTN1 siRNA #4		Bhadra et al., 2021
Sample 10-12	ERJ1 UTR siRNA #4		Bhadra et al., 2021
Sample 13-15	ERJ1 siRNA #6		Bhadra et al., 2021
**Orbi2514**	**Targeting of precursor polypeptides to the human ER**	**PXD011993**	
Sample 1-3	scr control siRNA		
Sample 4-6	SND2 siRNA #2		Tirincsi et al., 2022
Sample 7-9	SND2 siRNA #3		Tirincsi et al., 2022
Sample 10-12	WRB siRNA #3 + SND2 siRNA #2 = SW		Tirincsi et al., 2022
Sample 13-15	WRB siRNA #3 + SND2 siRNA #3 = SW		Tirincsi et al., 2022
Sample 16-18	HEK293 Mock CRISPR		Schorr et al., 2019
Sample 19-21	HEK293 SEC62 CRISPR		Schorr et al., 2019
Sample 22-24	HEK293 SEC63 CRISPR		Schorr et al., 2019
**Orbi2695**	**Targeting of proteins to the human ER**	**PXD12005**	
Sample 1-3	scr control siRNA		Zimmermann et al.,
Sample 4-6	PEX3 siRNA #1		Zimmermann et al.,
Sample 7-9	PEX3 siRNA #2		Zimmermann et al.,
Sample 10-12	control fibroblasts #3		Zimmermann et al.,
Sample 13-15	Zellweger patient fibroblasts DPEX3		Zimmermann et al.,

We note that the siRNA numbering may be different from the siRNA numbering in the respective reference and that in some siRNA experiments the scrambled (scr) control siRNA served as negative control for several targets and, possibly, different references. Therefore, in these latter cases no reference is given.

Table S2. Summary of clients as determined by MS and differential protein abundance analysis.

Clients of	Clients with			Clients with				
	SP	SP/I	SP/multi	TMH	TMH/II or III	TMH/multi	HP	TA
SR	***24***	***14***	**-**	***30***	***10***	***17***	***3***	**-**
Total	*54*							
% of total	*44.4 SP*			*55.6 TMH*				
% of total	*81.5 MP*							
% of TMH					*33.3*	*56.7*	*10*	-
% of total	*18.5sol*	*25.9*	-		*18.5*	*31.5*	*5.6*	-
Snd2	***16***	***8***	**2**	***39***	***5***	***26***	***1***	***7***
Total	*55*							
% of total	*29.1 SP*			*70.9 TMH*				
% of total	*89.1 MP*							
% of TMH					*12.8*	*66.7*	*2.6*	*17.9*
% of total	*10.9sol*	*14.5*	*3.6*		*9.1*	*47.3*	*1.8*	*12.7*
Snd2+Wrb	***13***	***5***	**2**	***30***	***3***	***21***	***1***	***5***
Total	*43*							
% of total	*30.2 SP*			*69.8 TMH*				
% of total	*86 MP*							
% of TMH					*10*	*70*	*3.3*	*16.7*
% of total	*14sol*	*11.6*	*4.7*		*7*	*48.8*	*2.3*	*11.6*
Wrb	***26***	***11***	**2**	***44***	***5***	***29***	***2***	***8***
Total	*70*							
% of total	*37.1 SP*			*62.9 TMH*				
% of total	*81.4 MP*							
% of TMH					*11.4*	*65.9*	*4.5*	*18.2*
% of total	*18.6sol*	*15.7*	*2.9*		*7.1*	*41.4*	*2.9*	*11.4*
PEX3	***28***	***5***	**1**	***22***	***9***	***7***	***2***	***4***
Total	*50*							
% of total	*56 SP*			*44 TMH*				
% of total	*56 MP*							
% of TMH					*40.9*	*31.8*	*9.1*	*18.2*
% of total	*44sol*	*10*	*2*		*18*	*14*	*4*	*8*
EMC	***11***	**-**	2	***48***	***1***	***45***	**-**	***2***
Total	*59*							
% of total	*18.6 SP*			*81.3 TMH*				
% of total	*84.7 MP*							
% of TMH					*2.1*	*93.8*	-	*4.2*
% of total	*15.3sol*	-	*3.4*		*1.7*	*76.3*	-	*3.4*

Clients of	Clients with			Clients with				
	SP	SP/I	SP/multi	TMH	TMH/II or III	TMH/multi	HP	TA
Sec61	***197***	*68*	*9*	***89***	***44***	***40***	**-**	***5***
Total	*286*							
% of total	*68.9 SP*			*31.1 TMH*				
% of total	*58.0 MP*							
% of TMH					*49.4*	*44.8*	-	*5.7*
% of total	*42.2sol*	*23.8*	*3.1*		*15.4*	*14.0*	-	*1.7*
TRAP	***67***	*15*	*7*	***57***	***13***	***42***	**-**	***2***
Total	*124*							
% of total	*54.0 SP*			*46.0 TMH*				
% of total	*63.7 MP*							
% of TMH					*22.8*	*73.7*	-	*3.5*
% of total	*36.3sol*	*12.1*	*5.6*		*10.5*	*33.9*	-	*1.6*
TRAM1	***13***	*2*	*2*	***17***	***10***	***5***	**-**	***2***
Total	*30*							
% of total	*43.3 SP*			*56.7 TMH*				
% of total	*70.0 MP*							
% of TMH					*58.8*	*29.4*	-	*11.8*
% of total	*30.0sol*	*6.7*	*6.7*		*33.3*	*16.7*	-	*6.7*
Sec62	***74***	*17*	*4*	***27***	***12***	***15***	**-**	**-**
Total	*101*							
% of total	*73.3 SP*			*26.7 TMH*				
% of total	*47.5 MP*							
% of TMH					*44.4*	*55.6*	-	-
% of total	*52.5sol*	*16.8*	*3.9*		*11.9*	*14.9*	-	-
Sec63	***32***	*11*	*3*	***35***	***13***	***21***	**-**	***1***
Total	*67*							
% of total	*47.8 SP*			*52.2 TMH*				
% of total	*73.1 MP*							
% of TMH					*37.1*	*60.0*	-	*2.9*
% of total	*26.9sol*	*16.4*	*4.5*		*12.1*	*35.6*	-	*1.7*
Sec62+Sec63	***19***	*8*	**-**	***11***	***4***	***7***	**-**	**-**
Total	*30*							
% of total	*63.3 SP*			*36.7 TMH*				
% of total	*63.3 MP*							
% of TMH					*36.4*	*63.6*	-	-
% of total	*36.7sol*	*26.7*	-		*13.3*	*23.3*	-	-

I, II, III, membrane protein type; HP, hairpin; MP, membrane protein; multi, multispanning membrane protein; sol, soluble; SP, signal peptide; TA, tail anchor; TMH, most N-terminal transmembrane domain; Zellweger, data from PEX3 deficient patient fibroblasts Zimmermann et al. [38]. Notably, sol refers to soluble proteins, in the cases of Wrb and Snd2 clients to the pool of the respective single depletion (Snd2 or Wrb) and the double depletion (Snd2+Wrb). Sec62+Sec63 represents the client overlap between the two single depletions.

Table S3. Characteristics of THM-containing clients of SR, Snd, Wrb, PEX3, and EMC.

Protein	TMDs	Type	TMH	TMH Sequence	Size	%	N	ERL-D	ΔG
**SR**									
TMTC3	multi	II	9-29	ITLIVGVVTACYWNSLFCGFV	914	1.8	+		1.675
B3GALTL	1	II	7-27	WWLLAPPALLALLTCSLAFGL	498	3.4	+		-0.935
SLC35B2	multi		5-25	WWAVVVLAAFPSLGAGGETPE	432	3.5			3.884
ERLIN2	1	II	4-24	LGAVVAVASSFFCASLFSAVH	339	4.1	+		1.947
REEP3	multi	HP	1-21	MVSWMISRAVVLVFGMLYPAY	255	4.3			0.697
SPTLC1	1		16-36	ALYEAPAYHLILEGILILWII	513	5.1			1.444
SLC16A3	multi	II	18-38	GGWGWAVLFGCFVITGFSYAF	465	6			-0.663
ZMPSTE24	multi	III	19-39	IFGAVLLFSWTVYLWETFLAQ	475	6.1			1.532
PDE3A	multi	II	61-81	LSSALCAGSLSFLLALLVRLV	1141	6.2		1	-0.598
ATP13A1	multi	II	67-87	VLPFAGLLYPAWLGAAAAGCW	1204	6.4	+		1.928
TMEM209	multi	II	28-48	VVLAWGLLNVSMAGMIYTEMT	561	6.8	+		1.535
PEX3*	1		16-36	CIFLGTVLGGVYILGKYGQKK	373	7			3.594
ABCC4	multi	II	93-113	LVLGIFTLIEESAKVIQPIFL	1325	7.8	+	2	5.118
ASPH	1	II	54-74	FFTWFMVIALLGVWTSVAVVW	758	8.4	+		-1.493
ATP2B1	multi	II	98-118	FLQLVWEALQDVTLIILEIAA	1220	8.9			4.019
ERGIC2	multi	II	34-54	GTVSLIAFTTMALLTIMEFSV	377	11.7			1.141
LNPEP	1	II	111-131	MVVCAFVIVVAVSVIMVIYLL	1025	11.8			-2.599
IKBIP	1		46-62	CLSLLSLGTCLGLAWFV	377	14.9	+		0.469
DEGS1	multi	II	41-61	PNLIWIIIMMVLTQLGAFYIV	323	15.8			-0.478
BST2	1	II	21-48	KLLLGIGILVLLIIVILGVPLIIFTIKA	180	17.2	+		-5.685
TMEM41B	multi	II	52-72	MSLLILVSIFLSAAFVMFLVY	291	21.3			-1.51
TVP23B	multi	II	34-53	PVASFFHLFFRVSAIIVYLL	205	21.5			1.886
CEPT1	multi	II	87-107	LITIIGLSINICTTILLVFYC	416	23.3	+		-0.476
ANO10	multi	II	208-228	IALYFGFLEYFTFALIPMAVI	660	32.7			1.133
SUN1	1	II	316-335	ICKFLVLLIPLFLLLAGLSL	785	41.5			-1.86
TOR1AIP2	1		215-235	FWSYGPVILVVLVVAVVASSV	470	47.9	+		-0.371
YIPF5	multi	II	125-145	TDLAGPMVFCLAFGATLLLAG	257	52.5			0.365
CAV1	1	HP	105-125	ALFGIPMALIWGIYFAILSFL	178	64.4			-0.726
ITPR3	multi	II	2203-2223	LWGSISFNLAVFINIIIAFFY	2671	82.9	+		0.138
ATL2	1	HP	477-497	TLFAVMFAMYIISGLTGFIGL	583	83.5	+		-0.074
**Snd2**									
PTGIS	1		1-20	MAWAALLGLLAALLLLLLLS	500	2.2			-3.435
MBOAT7	multi	III	9-29	LVVLLISIPIGFLFKKAGPGL	472	4	+		2.483
SLC39A7	multi	II	10-30	WVAVGLLTWATLGLLVAGLGG	469	4.3			0.193
SLC16A7	multi	II	16-36	GGWGWIVVGAAFISIGFSYAF	478	5.4			-0.035
GDPD4	multi	II	18-38	WVTFLGTGYWFFWSIFILSLA	520	5.4	+		0.185
SLC9A6	multi		28-48	LWLLLAVGVFDWAGASDGGGG	679	5.6	+		3.829
RHBDD2	multi		11-31	WCLCPEVPSATFFTALLSLLV	364	5.8			2.164
AGPAT5	multi		15-35	LLPSVVLLGTAPTYVLAWGVW	364	6.9			1.565
BCAP29	multi	III	7-27	AVATFLYAEIGLILIFCLPFI	241	7.1			-0.863
MYO9A	1		175-195	IYTYVGSILIVINPFKFLPIY	2548	7.3			1.571
SLC7A2	multi	II	38-59	DLIALGVGSTLGAGVYVLAGEV	658	7.3	+		2.693
ATP2C1	multi	II	71-91	LWKKYISQFKNPLIMLLLASA	919	8.8			4.913
POMK	1	II	21-43	VGLLLIMALMNTLLYLCLDHFFI	350	8.9	+		-0.095
ATG9A	multi	II	67-87	IFELMQFLFVVAFTTFLVSCV	839	9.2	+		-0.147
MXRA7	1		7-27	LLAALPALATALALLLAWLLV	170	10			-2.116
PLD3	1	II	39-59	VLLVLILAVVGFGALMTQLFL	490	10	+		-1.979
ATP12A	multi	II	102-123	EIVKFLKQMVGGFSILLWVGA	1093	10.3			3.561
CXCR4	multi	II	39-63	IFLPTIYSIIFLTGIVGNGLVILVM	352	13.9	+		-0.37
PTDSS2	multi	II	63-83	AHTLTVLFILTCTLGYVTLLE	487	15	+	1	-0.192
TMEM33	multi	II	32-52	LFTVYCSALFVLPLLGLHEAA	247	17	+		0.388
TMEM38B	multi	III	50-70	SWFTAMLHCFGGGILSCLLLA	291	20.6			0.584
TMEM41B	multi		52-72	MSLLILVSIFLSAAFVMFLVY	291	21.3			-1.951
REEP5	multi	HP	35-55	SFIALGVIGLVALYLVFGYGA	189	23.8			-1.133
SOAT1	multi	II	141-159	IYHMFIALLILFILSTLVV	550	27.5			-2.08
PRAF2	multi	II	42-62	LYYQTNYLLCFGIGLALAGYV	178	29.2			0.209
TMEM181	multi	II	153-173	HFVLVFVVFFICFGLTIFVGI	475	34.4			-2.195
TRPM7	multi	II	756-776	NSWYKVILSILVPPAILLLEY	1865	41.1		2	1.975
LEMD2	multi	II	213-233	LLLWASLGLLLVFLGILWVKM	503	44.3			-3.464

STEAP4	multi	II	196-216	LFPMWRFPFYLSAVLCVFLFF	459	44.9			-0.321
SEC62	multi	II	197-217	FVMGLILVIAVIAATLFPLWP	399	51.9			-1.555
SLC4A2	multi	II	708-731	CLAAVIFIYFAALSPAITFGGLLG	1241	57.9	+		-0.458
STX17	multi		229-249	LAALPVAGALIGGMVGGPIGL	302	79.1			2.128
C4orf3	1	TA	45-65	SYWLDLWLFILFDVVVFLFVYFL	65	84.6			-2.704
VAMP8	1	TA	76-96	MVLICVIVFIIILFIVLFAT	100	86			-4.429
VAMP4	1	TA	116-136	IKAIMALVAAILLLVIIILIV	141	89.4			-3.47
EMD	1	TA	223-243	VPLWGQLLLFLVFVIVLFFIY	254	91.7			-2.85
STX3	1	TA	264-284	LIIIIVLVVVLLGILALIIGLSV	289	94.8			-4.337
STX2	1	TA	265-288	WIIIAVSVVLVAIIALIIGLSVGK	288	95.5			-2.654
JPH1	1	TA	640-660	IMIVLVMLLNIGLAILFVHFL	661	98.3			-1.838

Snd2+Wrb

MBOAT7	multi	III	9-29	LVVLLISIPIGFLFKKAGPGL	472	4	+		2.483
SLC39A7	multi		10-30	WVAVGLLTWATLGLLVAGLGG	469	4.3			0.193
SLC16A7	multi	II	16-36	GGWGWIVVGAAFISIGFSYAF	478	5.4			-0.035
GDPD4	multi	II	18-38	WVTFLGTGYWFFWSIFILSLA	520	5.4	+	1	0.185
SLC9A6	multi		28-48	LWLLLAVGVFDWAGASDGGGG	679	5.6	+		3.829
RHBDD2	multi		11-31	WCLCPEVPSATFFTALLSLLV	364	5.8			2.164
AGPAT5	multi		15-35	LLPSVVLLGTAPTYVLAWGVW	364	6.9			1.565
BCAP29	multi	III	7-27	AVATFLYAEIGLILIFCLPFI	241	7.1			-0.863
ATP2C1	multi	II	71-91	LWKKYISQFKNPLIMLLLASA	919	8.8			4.913
POMK	1	II	21-43	VGLLLIMALMNTLLYLCLDHFFI	350	8.9	+		-0.095
ATG9A	multi	II	67-87	IFELMQFLFVVAFTTFLVSCV	839	9.2	+		-0.147
MXRA7	1		7-27	LLAALPALATALALLLAWLLV	170	10			-2.116
PLD3	1	II	39-59	VLLVLILAVVGFGALMTQLFL	490	10	+		-1.979
ATP12A	multi	II	102-123	EIVKFLKQMVGGFSILLWVGA	1039	10.3		1	3.561
CXCR4	multi	III	39-63	IFLPTIYSIIFLTGIVGNGLVILVM	352	13.9	+		-0.37
TMEM33	multi	II	32-52	LFTVYCSALFVLPLLGLHEAA	247	17	+	1	0.388
TMEM38B	multi	III	50-70	SWFTAMLHCFGGGILSCLLLA	291	20.6		1	0.584
REEP5	multi	HP	35-55	SFIALGVIGLVALYLVFGYGA	189	23.8			-1.133
SOAT1	multi	II	141-159	IYHMFIALLILFILSTLVV	550	27.5			-2.08
PRAF2	multi	II	42-62	LYYQTNYLLCFGIGLALAGYV	178	29.2			0.209
TMEM181	multi	II	153-173	HFVLVFVVFFICFGLTIFVGI	475	34.4			-2.195
LEMD2	multi	II	213-233	LLLWASLGLLLVFLGILWVKM	503	44.3		1	-3.464
STEAP4	multi	II	196-216	LFPMWRFPFYLSAVLCVFLFF	459	44.9			-0.321
SEC62	multi	II	197-217	FVMGLILVIAVIAATLFPLWP	399	51.9			-1.555
STX17	multi		229-249	LAALPVAGALIGGMVGGPIGL	302	79.1			2.128
C4orf3	1	TA	45-65	SYWLDLWLFILFDVVVFLFVYFL	65	84.6			-2.704
EMD	1	TA	223-243	VPLWGQLLLFLVFVIVLFFIY	254	91.7			-2.85
STX3	1	TA	264-284	LIIIIVLVVVLLGILALIIGLSV	289	94.8			-4.337
STX2	1	TA	265-288	WIIIAVSVVLVAIIALIIGLSVGK	288	95.5			-2.654
JPH1	1	TA	640-660	IMIVLVMLLNIGLAILFVHFL	661	98.3			-1.838

Wrb

MBOAT7	multi	III	9-29	LVVLLISIPIGFLFKKAGPGL	472	4	+		2.483
SLC39A7	multi		10-30	WVAVGLLTWATLGLLVAGLGG	469	4.3			0.193
REEP3	multi	HP	1-21	MVSWMISRAVVLVFGMLYPAY	255	4.3			0.697
EXT2	1	II	26-46	YITLFSIVLLGLIATGMFQFW	718	5			-1.428
GDPD4	multi	II	18-38	WVTFLGTGYWFFWSIFILSLA	520	5.4	+	1	0.185
SLC16A7	multi	II	16-36	GGWGWIVVGAAFISIGFSYAF	478	5.4			-0.035
SLC9A6	multi		28-48	LWLLLAVGVFDWAGASDGGGG	679	5.6	+		3.829
RHBDD2	multi		11-31	WCLCPEVPSATFFTALLSLLV	364	5.8			2.164
AGPAT5	multi		15-35	LLPSVVLLGTAPTYVLAWGVW	364	6.9			1.565
BCAP29	multi	III	7-27	AVATFLYAEIGLILIFCLPFI	241	7.1			-0.863
MFSD7	multi	II	30-50	WVFLLAISLLNCSNATLWLSF	559	7.2			0.335
NEU1	1		20-41	LGFWGGCRVWVFAAIFLLLSLAA	415	7.2	+		-1.467
MFSD10	multi	II	27-47	VVFLGLLLDLLAFTLLLPLLP	455	8.1			-1.459
ATP2C1	multi	II	71-91	LWKKYISQFKNPLIMLLLASA	919	8.8			4.913
POMK	1	II	21-43	VGLLLIMALMNTLLYLCLDHFFI	350	8.9	+		-0.095
ATG9A	multi	II	67-87	IFELMQFLFVVAFTTFLVSCV	839	9.2	+		-0.147
PTDSS1	multi	II	36-56	FFYRPHTITLLSFTIVSLMYF	473	9.7			0.972
MXRA7	1		7-27	LLAALPALATALALLLAWLLV	170	10			-2.116
PLD3	1	II	39-59	VLLVLILAVVGFGALMTQLFL	490	10	+		-1.979

ATP12A	multi	II	102-123	EIVKFLKQMVGGFSILLWVGA	1039	10.3		1	3.561
CXCR4	multi	III	39-63	IFLPTIYSIIFLTGIVGNGLVILVM	352	13.9	+		-0.37
TMEM33	multi	II	32-52	LFTVYCSALFVLPLLGLHEAA	247	17	+	1	0.388
TMEM38B	multi	III	50-70	SWFTAMLHCFGGGILSCLLLA	291	20.6		1	0.584
REEP5	multi	HP	35-55	SFIALGVIGLVALYLVFGYGA	189	23.8			-1.133
SOAT1	multi	II	141-159	IYHMFIALLILFILSTLVV	550	27.5			-2.08
PRAF2	multi	II	42-62	LYYQTNYLLCFGIGLALAGYV	178	29.2			0.209
SGPP1	multi	II	132-152	FCFGTELGNELFYILFFPFWI	441	32.2			2.661
TMEM181	multi	II	153-173	HFVLVFVVFFICFGLTIFVGI	475	34.4			-2.195
LBR	multi	II	212-232	VPGVFLIMFGLPVFLFLLLLM	615	36.1			-2.378
SPCS2	multi	II	87-107	ICTISCFFAIVALIWDYMHPF	226	42.9			1.255
LEMD2	multi	II	213-233	LLLWASLGLLLVFLGILWVKM	503	44.3		1	-3.464
STEAP4	multi	II	196-216	LFPMWRFPFYLSAVLCVFLFF	459	44.9			-0.321
SEC62	multi	II	197-217	FVMGLILVIAVIAATLFPLWP	399	51.9			-1.555
MARCH1	multi	II	155-175	IFCSVTFHVIAITCVVWSLYV	289	57.1			0.107
STX17	multi		229-249	LAALPVAGALIGGMVGGPIGL	302	79.1			2.128
ITPR3	multi	II	2203-2223	LWGSISFNLAVFINIIIAFFY	2671	82.9		1	0.138
C4orf3	1	TA	45-65	SYWLDLWLFILFDVVVFLFVYFL	65	84.6			-2.704
UBE2J2	1	TA	227-247	GLLGGALANLFVIVGFAAFAYTW	259	91.5			-1.019
EMD	1	TA	223-243	VPLWGQLLLFLVFVIVLFFIY	254	91.7			-2.850
FAR1*	1	TA	466-483	IRYGFNTILVILIWRIF	515	92.4			-0.255
STX3	1	TA	264-284	LIIIIVLVVVLLGILALIIGLSV	289	94.8			-4.337
STX2	1	TA	265-288	WIIIAVSVVLVAIIALIIGLSVGK	288	95.5			-2.654
GOLGA5	1	TA	699-719	VFVIIYMALLHLWVMIVLLTYTP	731	97			-2.261
JPH1	1	TA	640-660	IMIVLVMLLNIGLAILFVHFL	661	98.3			-1.838
**PEX3**									
AIFM2	1		7-27	VESGALHVVIVGGGFGGIAAA	373	4.6			2.886
COLEC12	1	II	38-58	FSIILLYILCALLTITVAILG	742	6.5	+		-2.162
CYBRD1	multi	II	12-32	LLGSALLVGFLSVIFALVWVL	286	7.7	+		-2.348
MAN1A1	1	II	42-62	FVLLLVFSAFITLCFGAIFFL	653	8	+		-2.612
ERMP1	multi	II	64-84	AGTGLSEVRAALGLALYLIAL	904	8.2	+	2	2.156
DHRS7B	1	II	18-38	FITSTAILPLLFGCLGVFGLF	325	8.6			-0.36
ENPP1	1	II	77-97	VLSLVLSVCVLTTILGCIFGL	925	9.4	+		-0.985
ABCD3*	multi	II	84-104	GYLVLIAVMLVSRTYCDVWMI	659	14.3	+	1	1.38
TMUB2	multi	III	36-56	VMVVAGVVVLILALVLAWLST	321	14.3			-2.382
SGCD	1	II	37-57	FFVLLLMILILVNLAMTIWIL	256	18.4			-3.808
PXMP2*	multi	II	31-51	LYPVLTKAATSGILSALGNFL	195	21			3.63
TMEM192	multi	II	47-67	TVIIVNLLWFIHLVFVVLAFL	271	21			-1.154
TMEM237	multi	II	227-247	MIGLFSHGFLAGCAVWNIVVI	408	58.1			1.917
PEX13*	1		227-251	AATSAKSWPIFLFFAVILGGPYLIW	403	58.8			0.039
TOR1AIP1	1		339-355	WLLPLIAALASGSFWFF	583	59.9			0.705
ATL1	1	HP	450-470	TLFVVIFITYVIAGVTGFIGL	558	82.4			-0.573
RTN3	multi	HP	864-887	LIMLLSLAAFSVISVVSYLILALL	1032	84.7			-2.436
VAMP3	1	TA	78-98	MWAIGITVLVIFIIIIIVWVV	100	88			-4.236
FAR1*	1	TA	466-483	IRYGFNTILVILIWRIFI	515	92.4			-0.668
STX6	1	TA	235-255	WCAIAILFAVLLVVLILFLVL	255	96.1	+		-5.096
ACBD5*	1		497-517	GVLTFAIIWPFIAQWLVYLYY	525	96.6			-0.121
CCDC136	1	TA	1130-1150	IFSLPLVGLVVISALLWCWWA	1154	98.8			-2.371
**EMC**									
PIEZO1	multi	II	5-25	VLGAVLYWLLLPCALLAACLL	2521	1	+	2	-1.673
ALG10	multi	II	7-27	YYFSAALSCTFLVSCLLFSAF	473	3.6			-0.56
LRRC8E	multi	II	23-43	WWDVLAEYLTVAMLMIGVFGC	796	4.1	+	1	1.41
GDE1	multi		4-24	WEDQGGLLGPFSFLLLVLLLV	331	4.2	+	1	1.467
SLC9A7	multi		22-42	LLLLPLLLGWGLRVAAAASAS	726	4.4	+		1.198
PLPP2	multi	II	5-25	WVFVLLDVLCLLVASLPFAIL	288	5.2		1	-1.092
SLC43A3	multi	II	17-37	LLECLGFAGVLFGWPSLVFVF	491	5.5	+		0.374
SLC44A1	multi	II	31-51	WLLLFILFCIGMGFICGFSIA	657	6.2		2	-2.846
SLC44A2[+]	multi	II	34-54	IICCVFLLLAIVGYVAVGIIA	706	6.2	+	1	-1.55
ATP11A	multi	II	62-82	PKNLFEQFRRVANFYFLIIFL	1134	6.3			6.227
ATP13A1[#]	multi	II	67-87	VLPFAGLLYPAWLGAAAAGCW	1204	6.4		1	1.928
BCAP31	multi	III	7-27	AVATFLYAEVFVVLLLCIPFI	246	6.9			-1.026
BCAP29	multi	III	7-27	AVATFLYAEIGLILIFCLPFI	241	7.1			-0.863

SLC7A1	multi	II	36-57	DLVALGVGSTLGAGVYVLAGAV	629	7.3	+		2.145
TMEM19	multi	II	15-35	MITNIVILSLIICISLAFWII	336	7.4		1	-2.479
ABCC4	multi	II	93-113	LVLGIFTLIEESAKVIQPIFL	1325	7.8		1	5.118
TMBIM6[#+]	multi	II	30-50	VYASFALCMFVAAAGAYVHMV	237	8.4			0.626
ZDHHC6	multi	II	25-45	IIALGVIAICSTMAMIDSVLW	413	8.5			0.605
ATP2B4	multi	II	93-113	FLELVWEALQDVTLIILEIAA	1170	8.8		1	4.199
SGPL1	1	III	41-61	LIAWSVVVWTLLIVWGYEFVF	568	9			-0.178
ATP2B1	multi	II	106-126	LQDVTLIILEIAAIVSLGLS	1220	9.5			1.33
ADCY9	multi	II	118-138	YALFYIGFACLLWSIYFAVHM	1353	9.5	+	1	-0.71
SLC6A6	multi	II	50-70	FVLSVAGGFVGLGNVWRFPYL	620	9.7			1.981
TM4SF1	multi	II	10-30	IGHSLVGLALLCIAANILLYF	202	9.9	+		-0.789
CD9	multi	II	13-33	LLFGFNFIFWLAGIAVLAIGL	228	10.1	+	1	-1.351
SLC1A3	multi	II	48-68	NAFVLLTVTAVIVGTILGFTL	542	10.7			-0.753
TMEM97	multi	II	10-30	VEWLLGLYFLSHIPITLFMDL	176	11.4			1.248
SLC38A5	multi	II	49-71	SFGMSVFNLSNAIMGSGILGLAY	472	12.7			3.308
DIRC2	multi	II	52-72	WLVLLLFSLLAFVQGLVWNTW	478	13			-1.075
ATP6V0C	multi	III	11-33	ASFFAVMGASAAMVFSALGAAYG	155	13.5			1.35
CXCR4	multi	III	39-63	IFLPTIYSIIFLTGIVGNGLVILVM	352	14.5	+		-0.37
MYADM	multi	II	41-61	LLQLVSTCVAFSLVASVGAWT	322	15.8			0.783
EBP	multi	III	29-49	WHILAGLFSVTGVLVVTTWLL	230	17			-0.37
SLC38A1	multi	II	75-97	MSVFNLSNAIMGSGILGLAFALA	487	17.5			1.72
ATP13A3[+]	multi	II	206-226	LLIKEVLNPFYIFQLFSVILW	1226	17.6			2.516
CLCN3	multi	II	209-232	MNYIMYIFWALSFAFLAVSLVKVF	818	26.9	+		-1.206
SOAT1[+]	multi	II	139-160	RTIYHMFIALLILFILSTLVVD	550	27.1			-2.49
ANO6[#]	multi	II	301-321	WLGYYTQMLLLAAVVGVACFL	910	34.2	+	2	-0.732
YIPF3	multi	II	149-169	IAGELYGPLMLVFTLVAILLH	350	45.4	+	1	0.961
TCIRG1	multi	II	386-404	VNPAPYTIITFPFLFAVMF	830	47.6		1	2.643
ATP6V0A1	multi	II	389-407	INPAPYTIITFPFLFAVM	837	47.7		1	3.192
SLC4A2[+]	multi	II	708-731	CLAAVIFIYFAALSPAITFGGLLG	1241	57.9	+		-0.458
ABCG2	multi	II	396-416	IAQIIVTVVLGLVIGAIYFGL	655	62	+	1	-1.275
TMEM199[+]	multi	II	146-166	LVITIFNFIVTVVAAFVCTYL	208	75			-0.893
MOSPD1	multi	II	159-179	SLLTVFLGVVCIAALMLPTLG	213	79.3			-1.284
ITPR1	multi	II	2283-2335	FWSSISFNLAVLMNLLVAFFYPF	2758	83.1		1	0.424
ZFPL1	1	TA	267-287	LLLLLGLLGFLALLALMSRLG	310	89.4			-3.489
FDFT1	1	TA	384-404	PIYLSFVMLLAALSWQYLTTL	417	94.5			0.069

Component (protein in **bold** face); Client protein; TMDs, number of transmembrane domains in client (including TMH); 1, single spanning; multi, multispanning; Type, membrane protein type; HP, hairpin; TA, tail anchor; TMH, most N-terminal transmembrane domain; TMH Sequence, primary structure of TMH; Size, number of amino acid residues of client; %, distribution of TMH in client (i.e. position of central amino acid residue of TMH in % of client primary structure); N, N-glycosylation; ERL-D, number of ER lumenal domains with a content of > 50 amino acid residues; ΔG, apparent Delta G for membrane insertion of TMHs (http://dgpred.cbr.su.se); highlights peroxisomal membrane proteins among the SR, Wrb and PEX3 clients; [#] highlights overlap with Sec61 clients; [+] highlights overlap with TRAP clients. Notably, Wrb and Snd2 refers to the pool of clients that were detected in the respective single depletion (Snd2 or Wrb) and the double depletion (Snd2+Wrb). MP types shown in red were determined by employing the most advanced prediction tool for this purpose (https://dtu.biolib.com/DeepTMHMM/).

Table S4. Characteristics of THM-containing clients of Sec61, TRAP, TRAM1, Sec62, and Sec63.

Protein	TMDs	Type	TMH	TMH Sequence	Size	%	N	ERL-D	ΔG
**Sec61**									
ERAP1	1	II	2-21	VFLPLKWSLATMSFLLSSLL	948	1.3	+		1.904
ANPEP	1	II	9-31	KSLGILGILLGVAAVCTIIALSVV	967	2.1	+		-1.669
SLC20A2	multi	III	6-26	YLWMVILGFIIAFILAFSVGA	652	2.5			-2.740
CNNM3	multi	II	11-27	LGWLFAALCLGNAAGEA	707	2.7	+	1	2.395
ABCC3	multi	III	33-53	TENPDLTPCFQNSLLAWVPCI	1527	2.8	+		7.305
GALNT2	1	II	7-24	MLLCFAFLWVLGIAYYMY	571	2.8			-1.679
GOLIM4	1	II	13-33	IFQTLLLLTVVFGFLYGAMLY	696	3.3	+		-1.635
GALNT1	1	II	9-28	VVLATSLIWVLLDMFLLLYF	559	3.4	+		-0.953
NCEH1	1	II	5-25	CVLLTALVALAAYYVYIPLPG	408	3.7	+		0.724
KIAA0319L	multi	II	30-50	SLYLFYTCFCFSVLWLSTDAS	1049	3.8	+	1	0.07
CLPTM1L	multi	II	11-31	LVVGVFVVYVVHTCWVMYGIV	538	3.9	+	1	1.222
MGAT1	1	II	7-29	AGLVLWGAILFVAWNALLLLFFW	445	4			-2.889
SLC30A1	multi	II	11-31	LLCMLALTFMFMVLEVVVSRV	507	4.1	+		-0.684
ERLIN2	1	II	4-24	LGAVVAVASSFFCASLFSAVH	339	4.1	+		1.947
SCARB1	multi	II	12-32	GALGVAGLLCAVLGAVMIVMV	509	4.3	+	1	-0.453
MGAT2	1	II	10-29	VLILTLVVAACGFVLWSSNG	447	4.5	+		0.142
SLC2A1	multi	II	12-33	LMLAVGGAVLGSLQFGYNTGVI	492	4.5	+		2.962
SLC39A9	multi	III	4-24	FISISLLSLAMLVGCYVAGII	307	4.6	+		-0.312
PIGG	multi	II	432-452	IYSMMVGTVVVLEVLTLLLLSV	983	5	+		-0.489
ERLIN1	1	III	8-28	VLVAAVVGLVAVLLYASIHKI	348	5.2	+		0.278
B3GAT3	1	II	8-28	VFLAYFLVSIAGLLYALVQLG	335	5.4	+		-0.348
HS2ST1	1	II	12-28	LQLLAVVAFAVAMLFLE	356	5.6	+		0.105
CASC4	1	II	15-35	SLVLVVLLVVIVVLAFNYWSI	436	5.7			-2.517
MOGS	1	II	39-59	STAGGVALAVVVLSLALGMSG	837	5.9	+		0.513
SLC16A3	multi		18-38	GGWGWAVLFGCFVITGFSYAF	465	6			-0.663
GOLM1	1	II	13-35	SPPLVLAALVACIIVLGFNYWIA	401	6	+		-1.479
IMPAD1	1	II	13-33	VAVFCLLGLGVLYHLYSGFLA	359	6.4	+		0.616
ATP13A1[#]	multi	II	67-87	VLPFAGLLYPAWLGAAAAGCW	1204	6.4	+	1	1.928
ATP11C	multi	II	67-85	FRRIANFYFLIIFLVQVTV	1132	6.7		2	0.357
NEU1	1		19-42	ILGFWGGCRVWVFAAIFLLLSLAA	415	7.2	+		-1.607
SLC7A2	multi	II	38-59	DLIALGVGSTLGAGVYVLAGEV	658	7.3	+		2.693
ERMP1	multi	II	64-84	AGTGLSEVRAALGLALYLIAL	904	8.2	+	2	2.156
ASPH	1	II	54-74	FFTWFMVIALLGVWTSVAVVW	758	8.4	+		-1.493
SLC25A40	multi	II	19-39	MLASCTGAILTSVIVTPLDVV	338	8.6			2.013
B4GALT1	1	II	25-44	LLVAVCALHLGVTLVYYLAG	398	8.8	+		0.011
TMEM38B	multi	III	20-33	FFDIAHYLVSVMAV	291	9.3			4.829
ERGIC3	multi	II	26-46	GGATVTIVSGLLMLLLFLSEL	383	9.4	+	1	0.098
GGCX	multi	II	61-81	PASLAVFRFLFGFLMVLDIPQ	758	9.4	+	2	1.521
CLN5	1	II	24-44	ASWCWALALLWLAVVPGWSRV	358	9.5	+		-0.338
PLD3	1	II	39-59	VLLVLILAVVGFGALMTQLFL	490	10	+		-1.979
LYRIC	1	III	49-69	GWVILVGTGALGLLLLFLLGY	582	10.1			-2.037
TFRC	1	II	68-88	YGTIAVIVFFLIGFMIGYLGY	760	10.3	+		-1.75
ECE1	1	II	69-89	LVVLVVLLAAGLVACLAALGI	770	10.3	+		-1.991
TMEM43	multi	II	32-52	TSGGMFVGLMAFLLSFYLIFT	400	10.5			-0.666
SLC12A2	multi	II	287-313	ESKGVVKFGWIKGVLVRCMLNIWGVM	1212	10.6		1	4.649
ITFG3	1	II	50-70	AAFFLSLFLCLFVVFVVSFVI	552	10.9	+		-2.892
ERGIC2	multi	II	34-54	GTVSLIAFTTMALLTIMEFSV	377	11.7		1	1.141
LNPEP	1	II	111-131	MVVCAFVIVVAVSVIMVIYLLPRCT	1025	11.8	+		-2.784
SLC12A4	multi	II	119-139	GTLMGVYLPCLQNIFGVILFL	1085	11.9	+	2	2.61
SPCS3	1	II	12-32	FAFSLSVMAALTFGCFITTAF	180	12.2	+		-0.437
GPRC5A	multi	III	34-54	AEAWGIVLETVATAGVVTSVA	357	12.3	+		3.613
P4HTM	1	II	61-81	FLVLMVFVHLYLGNVLALLLF	563	12.6	+		-0.816
ERGIC1	multi	II	27-47	IISICCCLFILFLFLSELTGF	290	12.8	+	1	-1.518
CANT1	1	II	45-62	VILTFFVGAAILWLLCSH	401	13.2	+		-0.542
MAN1B1	1	II	85-105	MILFLLAFLLFCGLLFYINLA	699	13.6			-3.78
GPX8	1		18-40	VFAVLLSIVLCTVTLFLLQLKFL	209	13.9			-2.349
CXCR4	multi	III	39-63	IFLPTIYSIIFLTGIVGNGLVILVM	352	14.2	+		-0.37
IKBIP	1	II	46-62	CLSLLSLGTCLGLAWFV	377	14.3	+		0.469
CD70	1	II	18-38	VLRAALVPLVAGLVICLVVCI	193	14.5	+		-0.925
SEC11A	1	II	17-36	QLYYQVLNFGMIVSSALMIW	185	14.6			2.116
VKORC1L1	multi		17-37	VARYAVCAAGILLSIYAYHVE	176	15.3			1.029

TMEM30A	multi		50-70	VLPIFFIIGLIFIPIGIGIFV	361	16.6	+	1	-1.855
TMBIM6[#]	multi	II	30-50	VYASFALCMFVAAAGAYVHMV	237	16.9			0.626
BST2	1	II	21-48	KLLLGIGILVLLIIVILGVPLIIFTIKA	180	18.9	+		-5.685
CKAP4	1	II	107-127	ALNFLFYLALVAAAAFSGWC	602	19.4			-0.004
ABHD12	1	II	75-95	ILFCVLGLYIAIPFLIKLCPGI	398	21.4	+		0.162
SCD	multi	II	73-93	WRNIILMSLLHLGALYGITLI	359	23.1			0.341
HTRA2	1	II	105-125	AWLAVALGAGGAVLLLLWGGG	458	25.1			-0.82
SUN2	1	II	213-233	FLWFLLPLLLLTCLTYGAWYF	717	31.1	+		-3.06
TOR4A	1	II	122-138	CLLLLVAIVGFQVLNAIENLDDNAQRY	423	31.9			5.049
FADS2	multi	II	132-152	FFLLLLAHIIALESIAWFTVF	444	32			-1.58
ANO6[#]	multi	II	301-321	WLGYYTQMLLLAAVVGVACFL	910	34.2	+	2	-0.732
SFXN1	multi	II	103-120	ITGCMMTFYRTTPAVLFW	322	34.8			4.764
TOR1AIP1	1		339-355	LLPLIAALASGSFWFF	583	38.6			1.307
SPCS2	multi	II	87-107	ICTISCFFAIVALIWDYMHPF	226	42.9			1.255
STEAP3	multi	II	208-228	LLPAWKVPTLLALGLFVCFYA	488	44.7	+		0.176
TOR1AIP2	1		215-235	FWSYGPVILVVLVVAVVASSV	470	47.9	+		-0.371
YIF1A	multi	II	139-159	LYIPTMAFITYVLLAGMALGI	293	50.9			-0.202
LEMD3	multi	II	475-495	MFLLTAACLFFLILGLTYLGM	911	53.2		1	-3,427
CLPTM1	multi	II	355-375	YLLALTIIVSIVHSVFEFLAF	669	54.6	+	1	0.339
DNAJB12	1	II	244-264	LGVFVQLMPILILILVSALSQ	375	67.7			0.462
ITPR3	multi	II	2203-2223	LWGSISFNLAVFINIIIAFFY	2671	82.9		1	0.138
FAR1	1	TA	466-483	IRYGFNTILVILIWRIF	515	92.4			-0.255
STX6	1	TA	235-255	VSHMTSDRRQWCAIAILFAVLLVVLILFLVL	255	96.1			-1.56
MOSPD2	1	TA	497-518	LLLSLTMLLLAFVTSFFYLLYS	518	97.9			-2.495
CDKAL1	1	TA	556-578	DCALRMSVGLALLGLLFAFFVKVY	579	97.9			-1.466
FNDC3B	1	TA	1182-1202	IIVLGFATLSILFAFILQYFLM	1204	99			-2.09

TRAP

SLC38A10	multi	II	4-24	AAASNWGLITNIVNSIVGVSV	1119	1.3			5.0
LMBRD2	multi	III	6-21	LGLEIVFVFFLALFLL	695	2	+		-1.255
FAM20B	1	II	7-25	VVLLAILLVIFIFTKVFLI	409	3.9	+		-2.577
SLC2A1	multi	II	12-33	LMLAVGGAVLGSLQFGYNTGVI	492	4.5	+		2.962
TPST2	1	II	9-25	LLAAGCALVLVLAVQLG	377	4.5	+		0.07
ARMC10	1		5-27	RGAGWVAAGLLLGAGACYCIYRL	343	4.7			-0.863
SLC2A10	multi	II	16-36	LLGGLTFGYELAVISGALLPL	541	4.8		1	1.57
WLS	multi	II	16-36	IVGGILLVFQIIAFLVGGLIA	541	4.8		1	-0.518
GLT8D1	1	II	8-28	IIILVLAVALFLLVLHHNFLS	371	4.9	+		-0.938
EXT2	1	II	26-46	YITLFSIVLLGLIATGMFQFW	718	5	+		-1.428
C1GALT1	1		7-29	LNFLTFLCGSAIGFLLCSQLFSI	363	5			-0.432
ARMCX6	1		7-27	VGWMAAGLMIGAGACYCVYKL	300	5.7			0.453
ATP2A3	multi	II	49-69	LWELVLEQFEDLLVRILLLAA	999	5.9			4.042
SLC44A2[+]	multi	II	34-54	IICCVFLLLAIVGYVAVGIIA	706	6.2	+	2	-1.55
SLC39A11	multi	III	12-32	LLGTFFTWGMTAAGAALVFVF	342	6.4			0.417
NEU1	1		19-42	ILGFWGGCRVWVFAAIFLLLSLAA	415	7.2	+		-1.607
CHRM2	multi	III	23-45	VVFIVLVAGSLSLVTIIGNILVM	466	7.3	+		0.049
CDIPT	multi	II	6-26	IFLFVPNLIGYARIVFAIISF	213	7.5			1.964
MFSD10	multi	II	27-47	VVFLGLLLDLLAFTLLLPLLP	455	8.1			-1.459
SLC22A18	multi	II	26-46	LLTYVLAATELTCLFMQFSI	424	8.5			1.373
SLC7A14	multi	II	58-78	ISLGVGSCVGTGMYVVSGLV	771	8.8	+		3.664
STT3B	multi	II	66-85	AGWQSLLSFTILFLAWLAGF	826	9.2	+	2	-0.173
CD63	multi	II	12-32	LLYVLLLAFCACAVGLIAVG	238	9.2	+	1	-2.096
ATP1A2	multi	II	86-106	EWVKFCRQLFGGFSILLWIGA	1020	9.4			2.551
LPCAT4	multi	II	40-62	CLLGALLAPIRVLLAFIVLFLLW	524	9.7	+		-2.51
TRAM1	multi	II	30-50	VAMVFLLGLMFEITAKASIIF	374	10.7	+		1.364
HM13	multi	III	32-52	IALAYGSLLLMALLPIFFGAL	377	11.1	+		-1.32
LNPEP	1	II	111-131	MVVCAFVIVVAVSVIMVIYLLPRCT	1025	11.8	+		-2.784
CHST14	1		40-60	LLPSMLMFAVIVASSGLLLMI	376	13.3	+		-0.887
TMEM205	multi	III	18-38	VLSGAWGMQMWVTFVSGFLLF	189	14.8			1.573
DEGS1	multi	II	41-61	PNLIWIIIMMVLTQLGAFYIV	323	15.8			-0.478
TMEM39A	multi	II	72-92	SLLFEFLFFIYLLVALFIQYI	488	16.8	+		-1.702
TMBIM6[+]	multi	II	30-50	VYASFALCMFVAAAGAYVHMV	237	16.9			0.626
ATP13A3[+]	multi	II	206-226	LLIKEVLNPFYIFQLFSVILW	1226	17.6			2.516
SGPP1	multi	II	133-152	CFGTELGNELFYILFFPFWI	441	20.4			2.995
TMEM189	multi	II	48-68	WCSVILCFSLIAHNLVHLLLL	271	21.4		1	0.867
KCNJ2	multi	II	82-106	RWMLVIFCLAFVLSWLFFGCVFWLI	427	22			-4.554

SGMS2	multi	II	80-100	IAFIYAVFNLVLTTVMITVV	365	24.7			0.258
TSPAN10	multi	II	79-99	IFLSNFPFSLLGLLALAIGL	355	25.1	+	1	0.631
TMEM223	multi	II	44-64	FTILGLFCAGQGVFWASMAV	202	26.7			1.206
SOAT1[+]	multi	II	139-160	RTIYHMFIALLILFILSTLVVD	550	27.1			-2.49
TMEM166	1		35-55	ALYFVSGVCIGLVLTLAALVI	152	29.6			-1.662
TMEM159	multi	II	44-61	YLDSHPFLAFTLLVFIVM	161	32.9			1.693
ANO10	multi	II	208-228	IALYFGFLEYFTFALIPMAVI	660	33			1.133
DAD1	multi	II	31-51	LLYILLTGALQFGYCLLVGTF	113	36.3			0.006
SPCS2	multi	II	87-107	ICTISCFFAIVALIWDYMHPF	226	42.9			1.255
PIGG	multi	II	432-452	IYSMMVGTVVVLEVLTLLLLSV	983	45	+		-0.489
TMEM261	multi	II	52-69	VLSGLGLMGAGGYVYWVA	116	50.9			2.007
SLC4A2[+]	multi	II	708-731	CLAAVIFIYFAALSPAITFGGLLG	1241	57.9	+		-0.458
PEX13	1		227-251	AATSAKSWPIFLFFAVILGGPYLIW	403	59.3			0.039
NOC4L	multi		297-317	ACDLGGALSLLALNGLFILI	516	59.5			0.828
TMEM199[+]	multi	II	146-166	LVITIFNFIVTVVAAFVCTYL	208	75			-0.893
STX17	multi		229-249	LAALPVAGALIGGMVGGPIGL	302	79.1			2.128
TMEM55B	multi	II	212-232	ICCFLLGLLLAVTATGLAFG	277	80.1			-1.224
WDR11	1		1127-1147	ALLVLLSLGCFFSVAETLHSM	1224	92.9			1.344
MOSPD2	1	TA	497-518	LLLSLTMLLLAFVTSFFYLLYS	518	97.9			-2.495
JPH2	1	TA	675-695	ILICMVILLNIGLAILFVHLL	696	98.4			-2.333

TRAM1

GALNT3	1	II	20-37	FWKLGAVIFFFIIVLVLM	633	3	+		-2.143
ERLIN2	1	II	4-24	LGAVVAVASSFFCASLFSAVH	339	4.1	+		1.947
NEU1	1		19-42	ILGFWGGCRVWVFAAIFLLLSLAA	415	7.2	+		-1.607
KCNN4	multi	II	29-49	LVLAGTGIGLMVLHAEMLWF	427	9.1			0.656
CLN5	1	II	24-44	ASWCWALALLWLAVVPGWSRV	358	9,5	+		-0.338
PLD3	1	II	39-59	VLLVLILAVVGFGALMTQLFL	490	10	+		-1.979
LYRIC	1	III	49-69	GWVILVGTGALGLLLLFLLGY	582	10.1			-2.037
LNPEP	1	II	111-131	MVVCAFVIVVAVSVIMVIYLLPRCT	1025	11.8	+		-2.784
P4HTM	1	II	61-81	FLVLMVFVHLYLGNVLALLLF	563	12.6	+		-0.816
SEC11A	1	II	17-36	QLYYQVLNFGMIVSSALMIW	185	14.6			2.116
DEGS1	multi	II	41-61	PNLIWIIIMMVLTQLGAFYIV	323	15.8			-0.478
PLP2	multi	II	25-45	GILLFAEIILCLVILICFSAS	152	23	+		-2.274
TMEM223	multi	II	44-64	FTILGLFCAGQGVFWASMAV	202	26.7			1.206
TOR4A	1		122-138	CLLLLVAIVGFQVLNAIENLDDNAQRY	423	30.7			5.049
STEAP3	multi	II	208-228	LLPAWKVPTLLALGLFVCFYA	488	44.7	+		0.176
BNIP3L	1	TA	188-208	VFIPSLFLSHVLALGLGIYIG	219	90.4			0.499
JPH1	1	TA	640-660	IMIVLVMLLNIGLAILFVHFL	661	98.3			-1.838

Sec62

PIEZO1	multi	II	5-25	VLGAVLYWLLLPCALLAACLL	2521	1	+	2	-1.673
PIGN	multi		2-24	LLFFTLGLLIHFVFFASIFDIYF	931	1.3	+	1	-2.669
PIGO	multi		4-24	ASVLLFLAWVCFLFYAGIALF	1089	1.3	+	1	-2.349
ERAP1	1	II	2-21	VFLPLKWSLATMSFLLSSLL	948	1.3	+		1.904
SLC20A2	multi	III	6-26	YLWMVILGFIIAFILAFSVGA	652	2.5			-2.740
B3GALTL	1	II	7-27	WWLLAPPALLALLTCSLAFGL	498	3.4	+		-0.935
MBOAT7	multi	III	6-22	WTYLVVLLISIPIGFLF	472	3.4	+		-0.795
FAM20B	1	II	7-25	VVLLAILLVIFIFTKVFLI	409	3.9	+		-2.577
ERLIN2	1	II	4-24	LGAVVAVASSFFCASLFSAVH	339	4.1	+		1.947
LRRC8C	multi	II	23-43	WWDVFTDYLSVAMLMIGVFGC	803	4.1	+	1	2.157
AGPAT4	multi	II	11-31	FLCHLVFCYVFIASGLIINTI	378	5.6		1	-0.023
MOGS	1	II	39-59	STAGGVALAVVVLSLALGMSG	837	5.9	+		0.513
UXS1	1	II	20-40	LLLGIALLAYVASVWGNFVNM	425	7.1	+		1.544
SLC7A2	multi	II	38-59	DLIALGVGSTLGAGVYVLAGEV	658	7.3	+		2.693
POMK	1	II	21-43	VGLLLIMALMNTLLYLCLDHFFI	350	8.9	+		-0.095
CLN5	1	II	24-44	ASWCWALALLWLAVVPGWSRV	358	9.5	+		-0.338
LNPEP	1	II	111-131	MVVCAFVIVVAVSVIMVIYLLPRCT	1025	11.8	+		-2.784
P4HTM	1	II	61-81	FLVLMVFVHLYLGNVLALLLF	563	12.6	+		-0.816
BST2	1	II	21-48	KLLLGIGILVLLIIVILGVPLIIFTIKA	180	18.9	+		-5.685
VANGL2	multi	II	109-129	VAAGATLALLSFLTPLAFLLL	521	22.8			-0.566
CNNM2	multi		251-271	FLLPFWLQVIFISLLLCLSGM	875	29.8	+		-1.562
AGPAT3	multi	II	125-145	ELLYVPLIGWTWYFLEIVFCK	376	35.9		1	2.308
YIPF2	multi	II	125-145	FWICATLAFVLAVTGNLTLVL	316	42.7			-0.977

TOR1AIP2	1		215-235	FWSYGPVILVVLVVAVVASSV	470	47.9	+		-0.371
ARL6IP6	multi	II	111-131	ILCSLLFAILLAFLLAIAYLI	226	53.5			-4.18
RNF5	multi	II	118-138	GGFHFSFGVGAFPFGFFTTVF	180	71.1			2.186
ITPR3	multi	II	2203-2223	LWGSISFNLAVFINIIIAFFY	2671	82.9		1	0.138

Sec63

TMTC3	multi	II	9-29	ITLIVGVVTACYWNSLFCGFV	914	2.1	+	2	1.675
SLC20A2	multi	III	6-26	YLWMVILGFIIAFILAFSVGA	652	2.5			-2.74
GALNT13	1	II	5-27	VYCKVVLATSLMWVVLVDVFLLLY	556	2.7	+		-0.402
FAM20B	1	II	7-25	VVLLAILLVIFIFTKVFLI	409	3.9	+		-2.577
STT3A	multi	II	18-38	LKLLILSMAAVLSFSTRLFAV	705	4	+	1	-0.285
LRRC8C	multi	II	23-43	WWDVFTDYLSVAMLMIGVFGC	803	4.1	+	1	2.157
LRRC8E	multi	II	23-43	WWDVLAEYLTVAMLMIGVFGC	796	4.1	+	1	1.41
ILVBL	1		13-33	LFPSFLLLACGTLVAALLGAA	510	4.5			-0.86
MGAT2	1	II	10-29	VLILTLVVAACGFVLWSSNG	447	4.5	+		0.142
GLT8D1	1		8-28	IIILVLAVALFLLVLHHNFLS	371	4.9	+		-0.938
AGPAT4	multi	II	11-31	FLCHLVFCYVFIASGLIINTI	378	5.6		1	-0.023
DGKE	1		22-42	LILWTLCSVLLPVFITFWCSL	567	5.6			-1.593
UXS1	1	II	20-40	LLLGIALLAYVASVWGNFVNM	425	7.1	+		1.544
ATP2C1	multi	II	71-91	LWKKYISQFKNPLIMLLLASA	919	8.8			4.913
GGCX	multi	II	61-81	PASLAVFRFLFGFLMVLDIPQ	758	9.4	+	2	1.521
CLN5	1	II	24-44	ASWCWALALLWLAVVPGWSRV	358	9.5	+		-0.338
NIPA1	multi	III	28-48	VSLGLGVAVVSSLVNGSTFVL	329	11.6			2.618
ELOVL1	multi	III	23-43	PLMGSPLLMTSILLTYVYFVL	279	11.8			0.248
APMAP	1	II	41-61	VTFLMLAVSLTVPLLGAMMLL	416	12.3	+		-0.556
MAN1B1	1	II	85-105	MILFLLAFLLFCGLLFYINLA	699	13.6			-3.78
IKBIP	1		46-62	CLSLLSLGTCLGLAWFV	377	13.8	+		0.469
BST2	1	II	21-48	KLLLGIGILVLLIIVILGVPLIIFTIKA	180	18.9	+		-5.685
VANGL2	multi	II	109-129	VAAGATLALLSFLTPLAFLLL	521	22.8			-0.566
ALG9	multi	II	136-156	ILVFYFLRCLLAFVSCICELY	618	23.6	+	1	-0.274
TMEM181	multi	II	153-173	HFVLVFVVFFICFGLTIFVGI	612	26.6			-2.195
UBIAD1	multi	II	83-103	LLVGCAVAVLAVHGAGNLVNT	338	27.5			2.583
CNNM2	multi	II	251-271	FLLPFWLQVIFISLLLCLSGM	875	29.8	+		-1.562
MARVELD2	multi	II	195-215	ILGVVELLLGAGVFACVTAYI	558	36.7			0.073
YIPF2	multi	II	125-145	FWICATLAFVLAVTGNLTLVL	316	42.7			-0.977
TEX2	multi	II	475-495	TLGFFIMCVYVYLILPLPHYV	1127	43			0.228
ARL6IP6	multi	II	111-131	ILCSLLFAILLAFLLAIAYLI	226	53.5			-4.18
TBC1D9B	1		668-688	LSWFLTLFLSVMPFESAVVIV	1233	55			0.956
UNC80	multi		2268-2288	PFVLQLFASVAPLLEFPDAAN	3258	69.9			4.606
TMEM199	multi	II	146-166	LVITIFNFIVTVVAAFVCTYL	208	75			-0.893
PARP16	1	TA	288-308	SHWFTVMISLYLLLLLIVSVI	322	92.5			-1.133

Sec62+Sec63

SLC20A2	multi	III	6-26	YLWMVILGFIIAFILAFSVGA	652	2.5			-2.740
FAM20B	1	II	7-25	VVLLAILLVIFIFTKVFLI	409	3.9	+		-2.577
LRRC8C	multi	II	23-43	WWDVFTDYLSVAMLMIGVFGC	803	4.1	+	1	2.157
AGPAT4	multi	II	11-31	FLCHLVFCYVFIASGLIINTI	378	5.6		1	-0.023
UXS1	1	II	20-40	LLLGIALLAYVASVWGNFVNM	425	7.1	+		1.544
CLN5	1	II	24-44	ASWCWALALLWLAVVPGWSRV	358	9.5	+		-0.338
BST2	1	II	21-48	KLLLGIGILVLLIIVILGVPLIIFTIKA	180	18.9	+		-5.685
VANGL2	multi	II	109-129	VAAGATLALLSFLTPLAFLLL	521	22.8			-0.566
CNNM2	multi	II	251-271	FLLPFWLQVIFISLLLCLSGM	875	29.8	+		-1.562
YIPF2	multi	II	125-145	FWICATLAFVLAVTGNLTLVL	316	42.7			-0.977
ARL6IP6	multi	II	111-131	ILCSLLFAILLAFLLAIAYLI	226	53.5			-4.180

Component (protein in **bold** face); Client protein; TMDs, number of transmembrane domains in client (including TMH); 1, single spanning; multi, multispanning; Type, membrane protein type; TA, tail anchor; TMH, most N-terminal transmembrane domain; TMH Sequence, primary structure of TMH; Size, number of amino acid residues of client; %, distribution of TMH in client (i.e. position of central amino acid residue of TMH in % of client primary structure); N, N-glycosylation; ERL-D, number of ER lumenal domains with a content of > 50 amino acid residues; ΔG, apparent Delta G for membrane insertion of TMHs (http://dgpred.cbr.su.se); [#] highlights overlap between Sec61 and EMC clients; [+] highlights overlap between TRAP and EMC clients. Notably, Sec62+Sec63 represents the client overlap. MP types shown in red were determined by employing the most advanced prediction tool for this purpose (https://dtu.biolib.com/DeepTMHMM/).

Table S5. Apparent ΔG values for membrane insertion of TMHs from MP clients of components for targeting to and/or insertion into the human ER membrane.

	**Component**	**Mean value**	**sem**
**TARGETING**	SR	0.501	0.397
	SND2	-0.416	0.37
	WRB	-0.287	0.303
	PEX3	-0.834	0.494
**INSERTION**	EMC	0.361	0.292
	SEC61	-0.073	0.23
	SEC62	-0.583	0.407
	SEC63	-0.183	0.374
	TRAP	0.095	0.254
	TRAM1	-0.273	0.497
**TA-TARGETING**	SND2	-3.239	0.358
	WRB	-2.523	0.438
	PEX3	-3.093	0.986
**TA-INSERTION**	EMC	-1.71	1.78
	SEC61	-1.573	0.378
	TRAP	-2.414	0.081
	TRAM1	-0.670	1.169

	**Component 1**	**Component 2**	***p* value**
**TARGETING**	SR	SND2	0.491
	SR	WRB	0.339
	SR	PEX3	**0.019**
	SND2	WRB	0.528
	SND2	PEX3	**0.062**
	WRB	PEX3	**0.058**
**INSERTION**	EMC	SEC61	0.552
	EMC	SEC62	**0.107**
	EMC	SEC63	**0.145**
	EMC	TRAP	0.829
	EMC	TRAM1	**0.149**
	SEC61	SEC62	0.471
	SEC61	SEC63	0.589
	SEC61	TRAP	0.724
	SEC61	TRAM1	0.463
	SEC62	SEC63	0.278
	SEC62	TRAP	**0.136**
	SEC62	TRAM1	1.000
	SEC63	TRAP	0.422
	SEC63	TRAM1	0.929
	TRAP	TRAM1	0.619
**TA-TARGETING**	SND2	WRB	0.285
	SND2	PEX3	0.465
	WRB	PEX3	0.715
**TA-INSERTION**	EMC	SEC61	0.655
	EMC	TRAP	0.655
	EMC	TRAM1	0.655
	SEC61	TRAP	**0.180**
	SEC61	TRAM1	0.655
	TRAP	TRAM1	**0.180**

The average apparent ΔG values for membrane insertion for TMHs of clients of four targeting components (SR, Snd2, Wrb, PEX3) and six membrane insertion components (Sec61, Sec62, Sec63, TRAMP, TRAM1, EMC) were determined (http://dgpred.cbr.su.se), taken from Tables S3 and S4, averaged and comparatively shown together with selected *p* values, as determined by the Wilcoxon signed-rank test using the SPSS software (version 27; IBM Corporation, Armonk, NY, USA).

Table S6. Protein transport components/*complexes* in HeLa cells and linked human diseases.

Component/subunit	Abundance [1]	Location [2]	Linked Human Diseases [3]
**for ER targeting**			
LRRC59 [4] (LRC59, p34) [5]	2480	ERM	
RRBP1 [4] (p180))	135	ERM	Hepatocellular Carcinoma, Colorectal Cancer
KTN1 (Kinectin 1)	263	ERM	
AEG-1 (LYRIC, MTDH)	575	ERM	
SRP [4,6]		C	
- SRP72	355		Aplasia, Myelodysplasia
- SRP68	197		
- SRP54	228		Neutropenia, Pancreas Insufficiency
- SRP19	33		
- SRP14	4295		Prostate Cancer
- SRP9	3436		Hepatocellular & Colorectal Carcinoma
- 7SL RNA			Breast-, Liver-, Lung-, Stomach-Cancer
SRP receptor		ERM	
- SRα (docking protein)	249		
- SRβ	173		
Snd1	unknown		
Snd receptor		ERM	
- Snd2 (TMEM208)	81		
- Snd3 (TMEM109)	49		
PEX19	80	C	Zellweger Syndrome
PEX3	103	ERM,PexM	Zellweger Syndrome
PEX16	9	ERM,PexM	Zellweger Syndrome
**for ER targeting & membrane insertion**			
Bag6 complex [4]		C	
- TRC35 (Get4)	171		CDG
- Ubl4A	177		
- Bag6 (Bat3)	133		Lung Cancer
SGTA	549	C	Breast Cancer, Lung Cancer
TRC40 (Asna1, Get3)	381	C	CDG
TA receptor		ERM	
- Caml (CAMLG, Get2)	5		CDG
- Wrb [7] (CHD5, Get1)	4		Congenital Heart Disease
**for ER membrane insertion**			
ERM protein complex		ERM	
- EMC1	124		Visual disorders
- EMC2	300		
- EMC3 [7]	270		
- EMC4	70		
- EMC5 (MMGT1)	35		
- EMC6 (TMEM93)	5		
- EMC7	247		
- EMC8	209		
- EMC9	1		
- EMC10	3		Developmental Delay
GEL complex		ERM	Glaucoma, Cerebrofaciothoracic Dysplasia
- TMCO1 [7]	2013		
- OPTI (C20Orf24, RAB5IF)	5		
- CCDC47 (Calumin)	193		
BOS complex [4]		ERM	
- Nicalin	99		
- TMEM147	21		
- NOMO	267		
PAT complex		ERM	
- PAT10 (Asterix)			
- CCDC47 (Calumin)	193		
**for membrane insertion & translocation**			
Sec61 complex [4]		ERM	
- Sec61α1	139		CVID, TKD, Neutropenia, PLD
- Sec61β	456		PLD, Colorectal Cancer
- Sec61γ	400		GBM, Hepatocellular Carcinoma

Alternative Sec61 complex		ERM	
- Sec61α2			
- Sec61β	456		
- Sec61γ	400		
Sec62/63 complex [4]		ERM	
- Sec62 (TLOC1)	26		Breast-, Prostate-, Cervix-, Lung-Cancer
- Sec63 (ERj2)	168		PLD, Colorectal Cancer
TRAM1 [4]	26	ERM	
TRAP complex [4]		ERM	
- TRAPα (SSR1)	568		
- TRAPβ (SSR2)			
- TRAPγ (SSR3)	1701		CDG, Hepatocellular Carcinoma
- TRAPδ (SSR4)	3212		CDG
**for folding & assembly**			
ER Chaperones			
- BiP (Grp78, HSPA5)	8253	ERL	HUS
- Calreticulin (CaBP3, ERp60)	14521	ERL	
- Calnexin [4]	7278	ERM	
- Grp170 (HYOU1)	923	ERL	
**for covalent modification**			
Oligosaccharyltransferase (OST-A) [4]		ERM	
- Ribophorin I (Rpn1)	1956		
- Ribophorin II (Rpn2)	527		
- OST48	273		CDG
- Dad1	464		
- OST4			
- TMEM258			
- Stt3A*	430		CDG
- DC2			
- Kcp2			
Oligosaccharyltransferase (OST-B)			
- Ribophorin I (Rpn1)	1956		
- Ribophorin II (Rpn2)	527		
- OST48	273		CDG
- Dad1	464		
- OST4			
- TMEM258			
- Stt3B*	150		CDG
- TUSC3			CDG
- MagT1	33		
Signal peptidase (SPC-A)		ERM	
- SPC12	2733		
- SPC18* (SEC11A)			
- SPC22/23	334		
- SPC25	94		
Signal peptidase (SPC-C)		ERM	
- SPC12	2733		
- SPC21* (SEC11C)			
- SPC22/23	334		
- SPC25	94		
GPI transamidase (GPI-T)		ERM	
- GPAA1	9		
- PIG-K	38		
- PIG-S	86		
- PIG-T	20		
- PIG-U	42		

The table was adapted from Lang et al. and Sicking et al. and updated [5,6]. [1] Abundance refers to the concentration (nM) in HeLa cells. [2] Localization refers to the functional intracellular localization(s) of the protein, i.e. C, Cytosol; ERL, ER lumen; ERM, ER membrane; PexM, Peroxisomal membrane. [3] Abbreviations for diseases: CDG, Congenital disorder of glycosylation; CVID, Common variable immunodeficiency; GBM, Glioblastoma; HUS, Hemolytic uremic syndrome; PLD, Polycystic liver disease; TKD, Tubulointerstitial kidney disease. [4] indicates ribosome association. [5] Alternative protein names are given in parentheses. [6] Complexes are indicated by italics. Abbreviations for protein names: BOS, Back of Sec61; EMC, ER membrane complex; GEL, GET- and EMC-like; GET, Guided entry of tail-anchored proteins; GPI, Glycosylphosphatidylinositol; SEC, involved in secretion; SND, SRP-independent; SPC, Signal peptidase; SR, SRP receptor; SRP, Signal recognition particle; SSR, Signal sequence receptor; TA, Tail anchor; TMEM, Transmembrane; TRAM, translocating chain-associating membrane (protein); TRAP, Translocon-associated protein; TRC, Transmembrane recognition complex. [7] indicates Oxa1 superfamily members.
